# Return of individual research results: What do participants prefer and expect?

**DOI:** 10.1371/journal.pone.0254153

**Published:** 2021-07-29

**Authors:** Sabina Sayeed, Robert Califf, Robert Green, Celeste Wong, Kenneth Mahaffey, Sanjiv Sam Gambhir, Jessica Mega, Bray Patrick-Lake, Kaylyn Frazier, Michael Pignone, Adrian Hernandez, Svati H. Shah, Alice C. Fan, Sarah Krüg, Terry Shaack, Scarlet Shore, Susie Spielman, Julie Eckstrand, Charlene A. Wong, on behalf of the Project Baseline Health Study Research Group

**Affiliations:** 1 Duke Clinical Research Institute, Duke University School of Medicine, Durham, North Carolina, United States of America; 2 Duke-National University of Singapore Medical School, Singapore City, Singapore; 3 Verily Life Sciences, South San Francisco, California, United States of America; 4 Brigham and Women’s Hospital, Broad Institute, Ariadne Labs, Harvard Medical School, Boston, Massachusetts, United States of America; 5 Department of Medicine, Stanford University School of Medicine, Stanford, California, United States of America; 6 Department of Radiology, Stanford University School of Medicine, Stanford, California, United States of America; 7 Evidation Health, Inc., San Mateo, California, United States of America; 8 Dell Medical School, University of Texas at Austin, Austin, Texas, United States of America; 9 Department of Medicine, Duke University Medical Center, Durham, North Carolina, United States of America; 10 Cancer101, New York, New York, United States of America; 11 California Health & Longevity Institute, Westlake Village, California, United States of America; 12 Stanford University School of Medicine, Stanford, California, United States of America; 13 Duke Clinical & Translational Science Institute, Duke University School of Medicine, Durham, North Carolina, United States of America; 14 Department of Pediatrics, Duke University School of Medicine, Durham, North Carolina, United States of America; 15 Duke-Margolis Center for Health Policy, Duke University, Durham, North Carolina, United States of America; Stellenbosch University Faculty of Medicine and Health Sciences, SOUTH AFRICA

## Abstract

Newer data platforms offer increased opportunity to share multidimensional health data with research participants, but the preferences of participants for which data to receive and how is evolving. Our objective is to describe the preferences and expectations of participants for the return of individual research results within Project Baseline Health Study (PBHS). The PBHS is an ongoing, multicenter, longitudinal cohort study with data from four initial enrollment sites. PBHS participants are recruited from the general population along with groups enriched for heart disease and cancer disease risk. Cross-sectional data on return of results were collected in 2017–2018 from an (1) in-person enrollment survey (n = 1,890), (2) benchmark online survey (n = 1,059), and (3) participant interviews (n = 21). The main outcomes included (1) preferences for type of information to be added next to returned results, (2) participant plans for sharing returned results with a non-study clinician, and (3) choice to opt-out of receiving genetic results. Results were compared by sociodemographic characteristics. Enrollment and benchmark survey respondents were 57.1% and 53.5% female, and 60.0% and 66.2% white, respectively. Participants preferred the following data types be added to returned results in the future: genetics (29.9%), heart imaging, (16.4%), study watch (15.8%), and microbiome (13.3%). Older adults (OR 0.60, 95% CI: 0.41–0.87) were less likely to want their genetic results returned next. Forty percent of participants reported that they would not share all returned results with their non–study clinicians. Black (OR 0.64, 95% CI 0.43–0.95) and Asian (OR 0.47, 95% CI 0.30–0.73) participants were less likely, and older participants more likely (OR 1.45–1.61), to plan to share all results with their clinician than their counterparts. At enrollment, 5.8% of participants opted out of receiving their genetics results. The study showed that substantial heterogeneity existed in participant’s preferences and expectations for return of results, and variations were related to sociodemographic characteristics.

## Introduction

The availability of broad health data generated through research, electronic health records, biobanks, and wearable devices has changed the health landscape in important ways [1,2]. By transforming our understanding of the constituents of health, these endeavors have paved the way for harnessing big data and developing a richer understanding of the transition from health to disease [3,4]. Central to these initiatives are longitudinal studies that allow for the collection and analysis of large volumes of data from participants. In addition to the logistical challenges inherent to such studies, their success is dependent upon the willingness of participants to engage in research, follow up for a predefined period, and derive a sense of value from their participation [5]. The motives for participant engagement, such as altruism or the desire to gain actionable health information, have emerged as important issues requiring characterization [6].

In recent years, calls for a more participant-centric research approach have included support for the belief that participants have a right to their health data and that investigators have an obligation to return research results to participants [7–10]. Prior work on returning single data types, such as imaging and genetic data, has shown promising but mixed results, with the opportunity to create value intermixed with cautionary notes of potential harms [11–15]. However, there remains a lack of clarity regarding the optimal mechanics of returning research results, with imaging and genetics paving the way while other data types remain in more nascent stages. Nevertheless, the enthusiasm for returning research results continues to grow, while several questions remain unanswered surrounding what to return, when, and how [16–18].

The Project Baseline Health Study (PBHS) is a multicenter, prospective, longitudinal cohort study that aims to characterize the multidimensional nature of health and disease by collecting and analyzing deep and diverse types of participant data, including imaging, digital device, and molecular data (projectbaseline.com/). The return of multiple types of individual research results has been an important value within PBHS since its inception in 2017. The objective of the current study is to describe the baseline preferences and expectations of participants on the return of individual research results within the PBHS. As one of the first studies to return multiple domains of participant data to several thousand participants, these findings can inform efforts to return individual research results in a way that creates value for participation.

## Methods

### Project baseline health study design

The PBHS is an ongoing, prospective, longitudinal cohort study across multiple sites; the initial sites were Stanford, Duke (two sites, one in Durham [Duke-Durham] and one in Kannapolis [Duke-Kannapolis]), and the California Health and Longevity Institute (CHLI) in the United States. The PBHS is a research partnership between Verily Life Sciences, Google, and the participating sites. The study was approved by the Western Institutional Review Board (WIRB) and local IRBs as required, and participants signed a consent form in order to participate in the study. The WIRB was the IRB for the study overall.

Research data collected from participants includes, but is not limited to, clinical and demographic characteristics, genetics, molecular, biochemical, imaging, behavioral, psychological, and physiological (e.g., a sleep sensor and study watch) data. Potential participants are identified through advertising, care provider recommendation, or community outreach activities. The goal is to recruit a population similar to the U.S. general census (standard cohort) with certain subcohorts enriched for the following disease risk areas: breast/ovarian cancer, lung cancer, and atherosclerotic cardiovascular disease. These enriched groups have an approximately 60% higher relative risk of disease compared to participants of the same age and sex, and were selected for enrichment because these are the leading causes of death in the U.S. and globally [19]. Participants have a two-day enrollment visit followed by annual visits for at least four years.

At enrollment, participants review and sign a consent form that indicates that participants will receive results from certain tests conducted as part of the PBHS. Participants are notified that the PBHS is a research study and not clinical care, and that it is not possible to fully opt-out of receiving some returned results. The consent form indicates that participants will have the option to decide whether they would like to receive genetic results. The return of research results policies and procedures were informed by a PBHS Return of Results Committee, which comprised clinicians, researchers, and participant advocates across the participating sites and external groups.

### Data collection

Data for the current study were collected from three sources: (1) enrollment in-person survey, (2) benchmark online survey, and (3) participant interviews. The enrollment in-person survey was administered during the participant’s first two-day visit. Data were collected by trained study staff for all participants enrolled between 3/30/17 and 11/30/18 on a case report form. The benchmark online survey was made available to participants one to 11 months post-enrollment through a digital app or website; data collected from 3/29/18 to 11/30/18 were included in this analysis. During the period between these two surveys, most participants received traditional clinical lab results (e.g., complete blood count) from the study, and some participants received results from imaging and onsite testing that were deemed to be critical or time sensitive (e.g., stress echocardiography results suggesting ischemia, or chest x-ray with large pulmonary nodule). Semi-structured interviews were conducted from 10/20/17 to 12/7/17 among a convenience sample of participants across sites before participants received any results. These interviews were conducted by the same study team member for approximately one hour with each study participant, and the interviews were later transcribed and analyzed independently by two other study team members.

### Outcomes

We examined three main outcomes: (1) preferences for type of result to be added next to the portfolio of returned results (benchmark survey), (2) participant plans for sharing returned results with a non-study clinician (benchmark survey), (3) choice to opt-out of receiving genetic results (enrollment survey). The survey items for the main outcomes were as follows: (1) Which of the following results would you be most interested in getting back next? (response options: genetics, microbiome, vital signs, physical assessment, cognitive assessments, heart imaging, eye imaging, sleep sensor data, study watch activity data), (2) Does the participant wish to opt out of genetic return of results? (response options: Yes or No), and (3) Which results from your site visits would you be likely to share with a doctor or nurse if you got them back? (response options: all results, results I do not understand, abnormal results, results my doctor or nurse is interested in having, results that are relevant for an existing health condition, I do not plan to share results with a doctor or nurse, I do not have access to a doctor or nurse).

Other outcomes in the benchmark survey included participant preferences regarding how results should be returned (i.e., phone call, email, mail or text), the benefits anticipated from receiving results (i.e., have ownership of my data, improve my health, identify my risks for disease, learn new or interesting things about my health, others, and I do not want my results or do not care about these benefits), and how participants feel about receiving results (i.e., excited to learn more about my health, nervous about the possibility of receiving an abnormal results, indifferent about getting my results backs, curious about the results, other, and none). All survey items are included in the Supplement.

The responses to all survey questions were mutually exclusive (i.e., participants could only select one answer) aside from the outcome question on sharing results, which allowed multiple answer selections.

The semi-structured interviews included questions on expectations and preferences for the return of research results, including their expectations on what they would receive and how it would be returned.

### Data analysis

Descriptive statistics are presented for survey responses, stratified by sociodemographic and clinical covariates: sex, age, study site, cohort (enriched or standard), self-reported race (white, black, Asian, or other) and ethnicity (Hispanic or non-Hispanic), income, and education level. For the three main outcomes, multivariable logistic regression models adjusted for these *a priori* selected sociodemographic and clinical covariates, based on the researcher’s knowledge and prior literature, are presented as odds ratios with 95% confidence intervals [7–15]. All quantitative analyses were conducted on Python (v2.7.1); a nominal p-value (i.e., unadjusted for multiple comparisons) of <0.05 was considered statistically significant.

Transcribed interview data were analyzed using an inductive thematic analysis approach [20]. Two authors (Sayeed, Wong) independently examined each transcript to identify and categorize relevant themes according to their frequency of appearance in the transcripts. The codebook was modified through an iterative process to account for newly emerging and unanticipated themes, consolidate themes, and revise code definitions. Content identified from the transcripts was sorted into different content sheets by themes and subthemes using Google Sheets, and coding disagreements were resolved via consensus. Illustrative quotes are presented to complement the quantitative results. Given the exploratory nature of this study, we did not apply adjustment for multiple comparisons.

## Results

The baseline enrollment survey included 1,890 participants, representing all those enrolled in the study at the time of analysis. The majority were female (57.1%), white (60.0%), and reported a yearly income over $50,000 (Table 1). Approximately one quarter (24.5%) had attended college and 26.2% had an advanced degree. Similar distributions were seen in the benchmark survey (N = 1,059, response rate 56.0%). In the subset who were interviewed (N = 21), 76% were white, 62% were advanced degree holders, and 52% were men. The overall study participants were similar to the general U.S. population with the exceptions of higher income and education distributions (Table 1).

**Table 1:** Project baseline health study participant demographics.

	Enrollment In-Person Survey	Benchmark Online Survey	Participant Interviews	General US Population
	(N = 1,890)	(N = 1,059)	(N = 21)	(%)
	n(%)	n(%)	n(%)	
**Sex**				
Female	1,079 (57.1)	567 (53.5)	10 (48)	(54.8)^a^
Male	811 (42.9)	492 (46.5)	11 (52)	(45.2) ^a^
**Age**				
18–39 years	625 (33.1)	382 (36.1)	9 (43)	(32.2) ^a^
40–59 years	673 (35.6)	377 (35.6.)	7 (33)	(31.4) ^a^
60+ years	592 (31.3)	300 (28.3)	5 (24)	(36.4) ^a^
**Study Site**				
Duke-Durham	403 (21.3)	165 (15.6)	5 (24)	N/A
Duke-Kannapolis	420 (22.2)	257 (24.3)	3 (14)	N/A
Stanford	700 (37.0)	419 (39.6)	6 (29)	N/A
CHLI	367 (19.4)	218 (20.6)	7 (33)	N/A
**Study Cohort**				
Enriched cohort^c^	714 (37.8)	386 (36.4)	4 (19)	N/A
Standard cohort	1176 (62.2)	673 (63.6)	17 (81)	N/A
**Race**				
White	1134 (60.0)	701 (66.2)	16 (76)	(80.4) ^a^
Black	355 (18.8)	135 (12.7)	2 (10)	(11.0)^a^
Asian	186 (9.8)	102 (9.6)	1 (5)	(5.2) ^a^
Other	215 (11.4)	121 (11.4)	2 (10)	(3.3) ^a^
**Ethnicity**				
Hispanic	239 (12.6)	144 (13.6)	1 (5)	(12.1) ^a^
Non-Hispanic	1651 (87.4)	915 (86.4)	20 (95)	(87.9) ^a^
**Annual Income**				
<$25,000	147 (7.8)	73 (6.9)	0 (0.0)	(20.2) ^b^
$25,000–50,000	196 (10.4)	119 (11.2)	1 (5)	(21.6) ^b^
$50,000–100,000	375 (19.8)	240 (22.7)	5 (24)	(29.0) ^b^
$100,000–200,000	371 (19.6)	277 (26.2)	8 (38)	(21.5) ^b^
>$200,000k	248 (13.1)	177 (16.7)	5 (24)	(7.7) ^b^
Declined to indicate	553 (29.3)	173 (16.3)	2 (10)	N/A
**Highest Education**				
High school or less	130 (6.9)	61 (5.8)	1 (5)	(39.9) ^b^
Some college	349 (18.5)	215 (20.3)	2 (10)	(28.6) ^b^
College degree	463 (24.5)	324 (30.6)	5 (24)	(20.0) ^b^
Advanced degree	496 (26.2)	345 32.6)	13 (62)	(11.4) ^b^
Declined to indicate	452 (23.9)	114 (10.8)	0 (0.0)	N/A

^a^ NHIS 2017.

^b^ US Census 2017.

^c^ Enriched cohorts include participants at higher risk of breast/ovarian cancer, lung cancer, and atherosclerotic cardiovascular disease. Of the 714 participants, 50.8% were at higher risk for atherosclerotic cardiovascular disease, 41.5% for breast/ovarian cancer risk, and 21.7% for lung cancer risk.

### Return of results preferences

When selecting among options of data types that participants preferred to receive after having received initial medical testing results, genetics (29.9%), heart imaging (16.4%), study watch (15.8%), and microbiome (13.3%) were the most common selections (Table 2). Participants over 60 years old (OR 0.60, 95% CI: 0.41–0.87) were less likely to want their genetic results returned next, while Hispanic participants (OR 1.55, 95% CI:1.01–2.39) were more likely than white participants to prefer their genetic data. Preferences differed by study site: participants at the Duke-Kannapolis site were more likely to want their study watch data next, while Stanford site participants had a strong preference for microbiome data compared to other sites. The majority of participants indicated that they would prefer to receive their results via phone (55.6%), followed by email (27.4%), text (13.9%), and mail (2.1%).

**Table 2:** Participant preferences on individual research results to return next (N = 1,059).

	Genetics next (n = 317)		Heart next (n = 174)		Watch next (n = 167)		Microbiome next (n = 141)	
	n (%)	OR^b^ (95% CI)	n (%)	OR^b^(95% CI)	n (%)	OR^b^ (95% CI)	n (%)	OR (95% CI)
**Sex**								
Female	176 (31.0)	Ref	99 (17.4)	Ref	83 (14.6)	Ref	73 (12.9)	Ref
Male	141 (28.7)	0.94 (0.71, 1.25)	75 (15.2)	0.79 (0.56, 1.11)	84 (17.0)	1.18 (0.83, 1.67)	68 (13.8)	0.96 (0.66, 1.40)
**Age**								
18–39	136 (35.6)**	Ref	57 (14.9)	Ref	62 (16.2)	Ref	54 (14.1)	Ref
40–59	112 (29.7)**	0.82 (0.59, 1.13)	64 (17.0)	1.13 (0.75, 1.71)	58 (15.4)	0.84 (0.56, 1.28)	53 (14.1)	0.98 (0.63, 1.52)
60+ years	69 (23.0)**	**0.60 (0.41, 0.87)**	53 (17.7)	1.26 (0.80, 1.99)	47 (15.7)	0.76 (0.48, 1.20)	34 (11.3)	0.69 (0.41, 1.15)
**Study site**								
Durham	48 (29.1)	1.19 (0.78, 1.81)	32 (19.4)	1.62 (0.98, 2.66)	30 (18.2)*	1.37 (0.82, 2.26)	18 (10.9)**	**0.52 (0.29, 0.94)**
Kannapolis	72 (28.0)	1.15 (0.78, 1.69)	44 (17.1)	1.28 (0.80, 2.04)	51 (19.8)*	**1.59 (1.01, 2.51)**	20 (7.8)**	**0.41 (0.23, 0.72)**
Stanford	119 (28.4)	Ref	37 (17.0)	Ref	62 (14.8)*	Ref	75 (17.9)**	Ref
CHLI	78 (35.8)	1.39 (0.96, 2.00)	61 (14.6)	1.19 (0.75, 1.90)	24 (14.8)*	0.73 (0.44, 1.23)	28 (12.8)**	**0.60 (0.36, 0.98)**
**Study cohort**								
Enriched cohort^a^	125 (32.4)	1.31 (0.98, 1.76)	54 (14.0)	0.72 (0.49, 1.05)	63 (16.3)	1.06 (0.74, 1.54)	34 (8.8)***	**0.50 (0.32, 0.77)**
Standard cohort	192 (28.5)	Ref	120 (17.8)	Ref	104 (15.5)	Ref	107 (15.9)***	Ref
**Race**								
White	209 (29.8)*	Ref	116 (16.5)	Ref	115 (16.4)*	Ref	99 (14.1)	Ref
Black	30 (22.2)*	0.66 (0.42, 1.05)	18 (13.3)	0.73 (0.42, 1.30)	20 (14.8)*	0.79 (0.46, 1.36)	20 (14.8)	1.28 (0.72, 2.26)
Asian	30 (29.4)*	0.93 (0.57, 1.51)	13 (12.7)	0.83 (0.43, 1.59)	22 (21.6)*	1.44 (0.83, 2.50)	11 (10.8)	**0.49 (0.24, 0.98)**
Other	48 (39.7)*	1.18 (0.75, 1.87)	27 (22.3)	1.46 (0.85, 2.52)	10 (8.2)*	0.56 (0.27, 1.17)	11 (9.1)	**0.44 (0.21, 0.90)**
**Ethnicity**								
Hispanic	61 (42.3)***	1.52 (0.99, 2.34)	28 (19.4)	1.13 (0.66, 1.93)	13 (9.0)*	0.66 (0.34, 1.28)	21 (14.6)	1.22 (0.68, 2.20)
Non-Hispanic	256 (28.0)***	Ref	146 (16.0)	Ref	154 (16.8)*	Ref	120 (13.1)	Ref
**Annual income**								
<$25,000	17 (23.3)	0.65 (0.32, 1.31)	13 (17.8)	1.49 (0.65, 3.45)	8 (11.0)	0.58 (0.23, 1.49)	7 (9.6)	0.93 (0.35, 2.44)
$25k-50k	41 (34.5)	0.92 (0.53, 1.58)	20 (16.8)	1.11 (0.54, 2.28)	13 (10.9)	0.61 (0.28, 1.32)	15 (12.6)	1.11 (0.53, 2.32)
$50k-100k	78 (32.5)	0.93 (0.59, 1.45)	29 (12.1)	0.83 (0.44, 1.56)	42 (17.5)	1.00 (0.57, 1.78)	31 (12.9)	0.95 (0.53, 1.70)
$100k-200k	77 (27.8)	0.78 (0.51, 1.18)	58 (20.9)	**1.82 (1.06, 3.13)**	48 (17.3)	1.11 (0.65, 1.87)	34 (12.3)	0.67 (0.39, 1.14)
>$200,000	59 (33.3)	Ref	22 (12.4)	Ref	27 (15.3)	Ref	34 (19.2)	Ref
Declined	45 (26.0)	0.83 (0.43, 1.61)	32 (18.5)	1.40 (0.62, 3.16)	29 (16.8)	1.14 (0.51, 2.54)	20 (11.6)	1.09 (0.47, 2.52)
**Highest education**								
≤ High school	17 (27.9)	0.80 (0.41, 1.55)	8 (13.1)	0.86 (0.36, 2.05)	6 (9.8)	0.93 (0.35, 2.44)	6 (9.8)*	0.59 (0.23, 1.56)
Some college	64 (29.8)	0.88 (0.58, 1.33)	43 (20.0)	1.51 (0.91, 2.50)	36 (16.7)	1.49 (0.88, 2.52)	18 (8.4)*	0.49 (0.27, 0.91)
College degree	102 (31.5)	0.98 (0.69, 1.37)	52 (16.0)	1.18 (0.77, 1.83)	60 (18.5)	1.56 (1.01, 2.40)	45 (13.9)*	0.71 (0.46, 1.10)
Advanced degree	105 (30.4)	Ref	50 (14.5)	Ref	47 (13.6)	Ref	61 (17.7)*	Ref
Declined	29 (25.4)	0.75 (0.36, 1.56)	21 (18.4)	1.21 (0.51, 2.84)	18 (15.8)	1.17 (0.49, 2.82)	11 (9.6)*	0.37 (0.14, 1.00)

Unadjusted analyses

*p<0.05

**p<0.01

***p<0.001. Bold text indicates significant adjusted results. The top 4 answer choices (i.e., genetics, heart, watch, microbiome) are displayed.

^a^ Enriched cohorts include participants at higher risk of breast/ovarian cancer, lung cancer, and atherosclerotic cardiovascular disease.

^b^ Multivariable models control for all covariates listed in table.

### Return of results expectations

When asked to describe how they felt about the possibility of receiving results back from their site visits, 54.9% of study participants said they were “excited to learn more about [their] health,” and 36.4% said they were “curious about the results.” Among survey respondents, 7.4% said they were “nervous” about receiving their results.

The most commonly selected expected benefits of receiving results were: “learning new things about my health” (72.5%), “improving my health” (72.0%), and “identifying my risks for disease” (70.1%). Fewer participants anticipated the benefit of having ownership of their data (38.1%).

With regard to the structured interview results (N = 21), while some participants desired and expected to receive all data related to their health returned to them, including in raw form, others expected only results that were actionable (Table 3). Other key themes from the structured interviews were expectations for results to be presented in a way that made them actionable and empowered participants to change their behavior. Participants also expressed that a motivator for their study participation was the desire to be able to track their health data, for example: *“Since it sounds like most of the tests will be repeated every year*, *it would be interesting to see if there’s any changes*, *or progression year-over-year*.*”*

**Table 3:** Qualitative participant expectations on the return of individual research results from structured interviews (N = 21).

**Actionability**	• **Desire to feel empowered to act on results**: *“Really*, *the whole thing is to empower me as an individual*. *That’s what I want*. *I want to be empowered to make decisions*. *Right*? *I know I have some bad habits*, *but I don’t know how bad*.*”* • **Results as a catalyst for behavior change**: *“Knowing about your own health gives you the opportunity to make simple lifestyle changes or choices…*. *The sleep study changed my consistency about getting enough sleep”* • **No expectations for clinical recommendations**: *“I expected to see the data with norms*. *Then if I saw something that was outside of the norm*, *I could decide to follow up with my medical practitioner*. *I wasn’t looking for any sort of recommendations*, *unless there was something that was egregiously wrong*.*”*
**Benchmarking of health data**	• **Comparisons to others like me**: *“I’m in my early 60s and so there are transitions in people’s physical health that they go through at different phases in their life*. *I’d like to see how my health trajectory looks compared to the other people in this age group*.*”* • **Tracking of personal health trends over time**: *“Since it sounds like most of the tests will be repeated every year*, *it would be interesting to see if there’s any changes*, *or progression year-over-year*.*”* • **Preparation for aging:** *“I’m so curious about aging*, *and how my measures would be changing over the five years that I’ll be studied here*. *What are the effects of aging on my body right now that might be reflected in any of these tests*?*”*
**Heterogeneity of expectations for return of results**	• **Desire no results**: *“I think it would be a huge logistical nightmare for you guys to find a way to deliver the test results without causing all sorts of extra labor*, *worry*, *fear and skew people’s behavior*. *If you’re really trying to look at how health happens in the transition between baseline and disease*, *then you kind of just have to be a fly on the wall*.*”* • **Desire all results**: *“I want all of it*, *whatever I can get my hands on*.*”* • **Desire raw data**: *“I have several friends that work in genetics who say ‘If you get genome data send that to us and we can help you figure yourself out*.*’ It’ll be really cool to have access to the raw data for a genome profile*.*“* • **Desire layman results**: *The results would be more in layman’s terms*. *Not just*, *‘Everything’s normal*,*’ but more information about how to either maintain [normal]*, *or if it’s not optimal*, *how to make it optimal*.*”*

### Sharing returned results

In the benchmark survey (n = 1059), 40% of participants (n = 426) reported not wanting to share all returned results with their own (i.e., non-study) clinicians (Table 4). Black (OR 0.64, 95% CI:0.43–0.95) and Asian (OR 0.47, 95% CI:0.30–0.73) participants were less likely to want to share all their results when compared to white participants. Compared with younger participants 18–39 years old, those in the 40–59 years (OR 1.59, 95% CI:1.16–2.17) and over 60 years (OR 1.45, 95% CI: 1.03–2.04) age groups were more likely to report wanting to share all their results.

**Table 4:** Participant preferences on sharing returned individual research results (n = 1,059)^a^.

	Share all results (n = 633)		Share abnormal results^b^ (n = 291)		Share results relevant to existing medical conditions^b^ (n = 210)		Share results I don’t understand^b^ (n = 168)	
	n (%)	OR^d^ (95% CI)	n (%)	OR^d^ (95% CI)	n (%)	OR^d^ (95% CI)	n (%)	OR (95% CI)
**Sex**								
Female	341 (60.1)	Ref	154 (68.1)	Ref	114 (50.4)	Ref	90 (39.8)	Ref
Male	292 (59.3)	1.00 (0.77, 1.29)	137 (68.5)	0.92 (0.57, 1.48)	96 (48.0)	0.89 (0.58, 1.35)	78 (39.0)	0.91 (0.59, 1.39)
**Age**								
18–39 years	191 (50.0)***	Ref	140 (73.3)	Ref	83 (43.5)	Ref	89 (46.6) *	Ref
40–59 years	247 (65.5)***	**1.61 (1.18, 2.20)**	83 (63.8)	0.59 (0.34, 1.03)	73 (56.2)	**1.64 (1.00, 2.67**)	44 (33.8) *	**0.52 (0.32, 0.87)**
60+ years	195 (65.0)***	**1.45 (1.03, 2.05)**	68 (64.8)	0.64 (0.34, 1.19)	54 (51.4)	1.20 (0.70, 2.07)	35 (33.3)*	**0.57 (0.32, 0.99)**
**Study site**								
Duke-Durham	99 (60.0) **	1.27 (0.85, 1.87)	51 (77.3)***	1.29 (0.60, 2.79)	32 (48.5)	0.87 (0.47, 1.61)	27 (40.9)	1.12 (0.60, 2.09)
Duke-Kannapolis	176 (68.5)**	**1.59 (1.11, 2.28)**	36 (44.4)***	**0.36 (0.19, 0.69)**	34 (42.0)	0.77 (0.42, 1.43)	26 (32.1)	0.83 (0.44, 1.56)
Stanford	236 (56.3)**	Ref	143 (78.1)***	Ref	96 (52.5)	Ref	71 (38.8)	Ref
CHLI	122 (56.0)**	1.06 (0.74, 1.49)	61 (63.5)***	**0.48 (0.26, 0.88**)	48 (50.0)	0.91 (0.53, 1.55)	44 (45.8)	1.30 (0.76, 2.23)
**Study cohort**								
Enriched cohort^c^	236 (61.1)	1.06 (0.80, 1.40)	94 (62.7)	0.85 (0.52, 1.39)	69 (46.0)	0.86 (0.55, 1.33)	55 (36.7)	0.86 (0.55, 1.35)
Standard cohort	397 (59.0)	Ref	197 (71.4)	Ref	141 (51.1)	Ref	113 (40.9)	Ref
**Race**								
White	449 (64.1)**	Ref	178 (70.6)*	Ref	133 (52.8)*	Ref	105 (41.7)	Ref
Black	73 (54.1)**	**0.64 (0.43, 0.95)**	33 (53.2)*	0.51 (0.25, 1.01)	24 (38.7)*	0.58 (0.31, 1.10)	26 (41.9)	1.24 (0.65, 2.37)
Asian	43 (42.2)**	**0.47 (0.30, 0.73)**	45 (76.3)*	0.76 (0.36, 1.61)	34 (57.6)*	1.17 (0.62, 2.19)	20 (33.9)	0.64 (0.33, 1.24)
Other	68 (56.2)**	1.01 (0.64, 1.57)	35 (66.0)*	0.81 (0.38, 1.73)	19 (35.8)*	0.56 (0.28, 1.10)	17 (32.1)	**0.45 (0.22, 0.92**)
**Ethnicity**								
Hispanic	74 (51.4)*	0.70 (0.46, 1.07)	47 (67.1)	0.93 (0.46, 1.86)	28 (40.0)	0.74 (0.40, 1.37)	33 (47.1)	1.65 (0.88, 3.10)
Non-Hispanic	559 (61.1)*	Ref	244 (68.5)	Ref	182 (51.1)	Ref	135 (37.9)	Ref
**Annual income**								
<$25,000	45 (61.6)	0.68 (0.36, 1.29)	12 (42.9)***	0.84 (0.25, 2.77)	13 (46.4)	1.06 (0.35, 3.21)	6 (21.4)	0.30 (0.09, 1.02)
$25,000–50,000	65 (54.6)	**0.56 (0.33, 0.95)**	35 (64.8)***	0.95 (0.36, 2.47)	27 (50.0)	1.25 (0.53, 2.97)	22 (40.7)	0.59 (0.24, 1.42)
$50,000–100,000	140 (58.3)	**0.63 (0.41, 0.98)**	69 (69.0)***	1.04 (0.46, 2.35)	49 (49.0)	0.92 (0.45, 1.90)	37 (37.0)	0.64 (0.31, 1.35)
$100,000–200,000	162(58.5)	0.67 (0.45, 1.01)	93 (80.9)***	1.90 (0.85, 4.21)	55 (47.8)	0.74 (0.38, 1.45)	50 (43.5)	0.89 (0.45, 1.76)
>$200,000	118 (66.7)	Ref	43 (72.9)***	Ref	35 (59.3)	Ref	24 (40.7)	Ref
Declined	103 (59.5)	0.60 (0.32, 1.12)	39 (55.7)***	0.55 (0.19, 1.60)	31 (44.3)	1.11 (0.41, 3.01)	29 (41.4)	0.82 (0.30, 2.24)
≤ High school	35 (57.4)	0.87 (0.47, 1.61)	9 (34.6)***	**0.24 (0.08, 0.69)**	13 (50.0)	0.98 (0.37, 2.63)	9 (34.6)	1.20 (0.42, 3.39)
Some college	134 (62.3)	1.12 (0.76, 1.67)	45 (55.6)***	0.60 (0.30, 1.22)	31 (38.3)	0.61 (0.32, 1.15)	31 (38.3)	1.15 (0.60, 2.22)
College degree	191 (59.0)	1.02 (0.73, 1.41)	98 (73.7)***	0.82 (0.45, 1.50)	70 (52.6)	0.99 (0.60, 1.64)	54 (40.6)	1.10 (0.60, 1.69)
Advanced degree	204 (59.1)	Ref	110 (78.0)***	Ref	78 (55.3)	Ref	56 (39.7)	Ref
Declined	69 (60.5)	1.33 (0.68, 2.59)	29 (64.4)***	1.13 (0.39, 3.31)	18 (40.0)	0.53 (0.19, 1.45)	18 (40.0)	0.87 (0.32, 2.40)

Of the 426 participants not wanting to share all results, 27.4% said they would share abnormal results, 19.8% said they would share results relevant to existing medical conditions, and 15.9% would share results they do not understand (Table 4). Compared to participants with advanced degrees, those with a high school diploma or less were less likely to report wanting to share abnormal results (OR 0.24, 95% CI: 0.08–0.69). Younger participants were more likely to report wanting to share results they did not understand than their older counterparts. Differences were noted by study site; participants at Duke-Kannapolis were more likely to report wanting to share all results and less likely to share abnormal results.

Interviewed participants stated that they did not expect clinical recommendations with their returned results, though they did express a desire to see their data benchmarked against normative data to inform whether they should see their own non-study clinician: *“I expected to see the data with norms*. *Then if I saw something that was outside of the norm*, *I could decide to follow up with my medical practitioner*. *I wasn’t looking for any sort of recommendations*, *unless there was something that was egregiously wrong”* (Table 3).

### Genetics opt-out

At enrollment, 5.5% of participants opted-out of receiving their genetics results (Table 5). Participants at the Duke-Durham (OR 8.62, 95% CI:4.44–16.73) and CHLI (OR 2.29, 95% CI: 1.06–4.96) sites or those with a high school education or less (OR 3.37, 95% CI:1.44–7.91) were more likely to opt out of receiving their genetic results.

**Table 5:** Participants choices on opting out from the return of individual genetics research results (N = 1,890).

	Opted-Out of Genetics Results Return n (%)	OR (95% CI)
**Overall**	104 (5.5)	N/A
**Sex**		
Female	63 (5.8)	Ref
Male	41 (5.1)	0.84 (0.54, 1.30)
**Age**		
18–39 years	30 (4.8)	Ref
40–59 years	48 (7.1)	1.21 (0.73, 2.01)
60+ years	26 (4.4)	1.10 (0.60, 1.99)
**Study Site**		
Duke-Durham	65 (16.1)	**8.62 (4.44, 16.73)**
Duke-Kannapolis	10 (2.4)	1.05 (0.44, 2.54)
Stanford	13 (1.9)	Ref
CHLI	16 (4.4)	**2.29 (1.06, 4.96**)
**Study Cohort**		
Enriched cohort^a^	49 (6.9)	1.24 (0.79, 1.94)
Standard cohort	55 (4.7)	Ref
**Race**		
White	50 (4.4)	Ref
Black	37 (10.4)	1.22 (0.72, 2.07)
Asian	9 (4.8)	1.32 (0.59, 2.92)
Other	8 (3.7)	0.74 (0.31, 1.76)
**Ethnicity**		
Hispanic	9 (3.8)	1.07 (0.46, 2.47)
Non-Hispanic	95 (5.8)	Ref
**Annual Income**		
<$25,000	9 (6.1)	0.36 (0.12, 1.08)
$25,000–50,000	20 (10.2)	0.91 (0.37, 2.28)
$50,000–100,000	18 (4.8)	0.48 (0.20, 1.15)
$100,000–200,000	14 (3.8)	0.62 (0.27, 1.44)
>$200,000	11 (4.4)	Ref
Declined to indicate	32 (5.8)	0.35 (0.09, 1.34)
**Highest Education**		
High school or less	16 (13.3)	**3.37 (1.44, 7.91)**
Some college	25 (7.2)	1.79 (0.88, 3.64)
College degree	13 (2.8)	0.77 (0.37, 1.60)
Advanced degree	21 (4.2)	Ref
Declined to indicate	29 (6.4)	2.39 (0.64, 8.89)

Bold text indicates significant adjusted results.

^a^ Enriched cohorts include participants at higher risk of breast/ovarian cancer, lung cancer, and atherosclerotic cardiovascular disease.

## Discussion

In a diverse sample of participants within the Project Baseline Health Study across four U.S. sites, individuals were excited and curious about receiving individual research results across various types of data streams. The majority of participants elected to receive their genetics results, while perhaps surprisingly, over a third said that they would not share all their results with their own clinicians. Participant expectations were heterogeneous, with younger participants preferring to receive their genetic results returned next while other participant preferences differed by sociodemographic characteristics.

Most participants in the PBHS were excited and curious to learn more about their health through the return of research results, mirroring previously described eagerness and curiosity about returned research results [21–23]. A growing literature recognizes that returning results serves a two-fold purpose: participants’ enthusiasm for returned results can help maintain their engagement for meeting the scientific goals of a study, while researchers can fulfill the increasingly accepted ethical obligation to share participants’ personal health data with them. Though the processes for returning individual results are in nascent stages of development, the scientific community has begun convening groups to outline recommendations and frameworks to guide the field, such as the proposed criteria of validity and actionability for deciding what to return [24,25]. Our qualitative results support the need for continued development of processes to return research results and highlights the importance of asking participants about their preferences and expectations early in the study protocol. Determining what is actionable and how to share data to facilitate appropriate action will require further work, especially when considering non-standard of care research tests for which there is no precedent consensus [24].

While participants were enthusiastic about receiving research results, 40% of survey respondents reported not planning to share all of their results with their non-study clinicians. Participants in the PBHS are voluntarily consenting to share their personal health data for research purposes, so their choice to not share all of their returned data with clinicians was surprising. Lower enthusiasm for sharing results with clinicians among black, Asian, and lower income participants may reflect differing levels of trust in healthcare and research, and suggests a need to monitor the impact of returning results on long-standing and continued health inequities, many of which have been exacerbated during the COVID-19 pandemic [26,27]. Moreover, prior work has hinted at mixed readiness among clinicians to receive research results; concerns include inadequate physician time for processing large quantities of research information [28–31]. Some participants stated they would only share abnormal results or results relevant to existing medical conditions, which could reduce the burden placed upon clinician time. Other strategies include co-designing processes with clinicians to understand how to incorporate returned research results into clinical workflows, such as within electronic medical records or by utilizing other team members (e.g., genetics counselors) [32,33].

The diversity of participant preferences and expectations will be a key challenge to returning research results in large clinical studies [34]. Participants in this survey differed not only in how much data they wanted returned, but also in the types of data they wanted to access. The heterogeneity of participant expectations on receiving results is similar to the diversity of patient preferences seen in clinical care, including differences seen by race and ethnicity [35–37]. Technology and data science advances can support individualizing how data are returned to participants, such as allowing participants to toggle result reports on and off or to select preferences on how their results are returned [11,38].

While participant preferences should be considered, there may be situations in which not returning results would be ethically inappropriate (e.g., a life-threatening finding with treatment options) where the duty to warn may come into conflict with participant wishes. The line is poorly defined between research participant autonomy to receive what they deem desirable, and the responsibility of researchers to highlight for participants the implications of clinically important results. Delineating what should be responsibly returned is even more difficult for results derived from non-standard data types, such as wearable device data or metabolomic results, where the limits of normalcy, or even the evidence for any sort of benefit, are ill-defined. Additionally, characterizing the ethical issues and complexities surrounding sharing potentially life-threatening results with relatives will also require further work [39]. In all cases, the importance of clearly explaining to participants the implications of choosing whether to receive individual research results cannot be overstated.

The results of this study should be interpreted in the context of several limitations. While the PBHS sample for this analysis included a diverse group of participants from multiple U.S. sites, the preferences and expectations of participants who chose to join this study may not be representative of research participants in different studies or other segments of the general population. While our study population was well matched to the overall U.S. population in most regards, differences were seen in income and educational levels, particularly among the interviewed subsample with higher proportions of white and highly educated individuals. Examining preferences by participant characteristics, including race and ethnicity, is critical given the historic and persistent racial and ethnic disparities in the quality and access to health care in the United States [40]. Despite these notable differences, interviewees in-depth insights provide richness to the quantitative results. Finally, the preferences and expectations described in this study were collected before participants had received results in most cases; participants’ perspectives and planned actions, such as sharing results with their clinicians, may change as results are returned in the study.

The novelty of the PBHS includes the commitment to asking participants directly what research results they would like to see returned and returning individual research results as a core element of the research endeavor. These early findings on the participants’ preferences and expectations for receiving research results will help the PBHS investigators refine the process. These data may also inform the broader research community in discussions about policies and practices that will promote a culture of partnership between researchers and study participants. The need for such partnerships will be accelerated as learning health systems align science, data, and clinical care [41], and biobanks accumulate and test a wider array of samples [42].

In the midst of growing rhetoric to free data, the diversity of participant perspectives and the nascent stage of returning research results at scale underscores the challenges ahead. These findings among a national cohort of participants in the PBHS provide an empirical basis for how research data can and should be returned in ways that may add impact and value to healthcare and research participation. While general trends in preferences exist as a function of age, race, ethnicity, sex, and geography, an important realization is that there is no substitute for asking people about their preferences.

## Supporting information

S1 File Return of individual research results and data dictionary. (DOCX)

S1 Data Plos one benchmark survey data (pre-RoR). (XLSX)

## References

[1] Alyass A, Turcotte M, Meyre D. From big data analysis to personalized medicine for all: challenges and opportunities. BMC medical genomics. 2015;8:33. Epub 2015/06/27. doi: 10.1186/s12920-015-0108-y ; PubMed Central PMCID: PMC4482045. 26112054 PMC4482045

[2] Beckmann JS, Lew D. Reconciling evidence-based medicine and precision medicine in the era of big data: challenges and opportunities. Genome Medicine. 2016;8(1):134. doi: 10.1186/s13073-016-0388-7 27993174 PMC5165712

[3] Tebani A, Afonso C, Marret S, Bekri S. Omics-Based Strategies in Precision Medicine: Toward a Paradigm Shift in Inborn Errors of Metabolism Investigations. International journal of molecular sciences. 2016;17(9). Epub 2016/09/21. doi: 10.3390/ijms17091555 ; PubMed Central PMCID: PMC5037827. 27649151 PMC5037827

[4] Sancesario GM, Bernardini S. Alzheimer’s disease in the omics era. Clinical biochemistry. 2018;59:9–16. Epub 2018/06/20. doi: 10.1016/j.clinbiochem.2018.06.011 . 29920246

[5] Kaufman D, Murphy J, Scott J, Hudson K. Subjects matter: a survey of public opinions about a large genetic cohort study. Genetics in medicine: official journal of the American College of Medical Genetics. 2008;10(11):831–9. Epub 2008/11/18. doi: 10.1097/GIM.0b013e31818bb3ab . 19011407

[6] Renegar G, Webster CJ, Stuerzebecher S, Harty L, Ide SE, Balkite B, et al. Returning genetic research results to individuals: points-to-consider. Bioethics. 2006;20(1):24–36. Epub 2006/05/10. doi: 10.1111/j.1467-8519.2006.00473.x . 16680905

[7] Knoppers BM, Joly Y, Simard J, Durocher F. The emergence of an ethical duty to disclose genetic research results: international perspectives. European Journal Of Human Genetics. 2006;14:1322. doi: 10.1038/sj.ejhg.5201690 16868560

[8] Faucett WA, Davis FD. How Geisinger made the case for an institutional duty to return genomic results to biobank participants. Applied & translational genomics. 2016;8:33–5. Epub 2016/04/06. doi: 10.1016/j.atg.2016.01.003 ; PubMed Central PMCID: PMC4796707. 27047758 PMC4796707

[9] Ottman R, Freyer C, Mefford HC, Poduri A, Lowenstein DH. Return of individual results in epilepsy genomic research: A view from the field. Epilepsia. 2018;59(9):1635–42. Epub 2018/08/12. doi: 10.1111/epi.14530 ; PubMed Central PMCID: PMC6119474. 30098010 PMC6119474

[10] Wolf SM. Return of individual research results and incidental findings: facing the challenges of translational science. Annual review of genomics and human genetics. 2013;14:557–77. Epub 2013/07/24. doi: 10.1146/annurev-genom-091212-153506 ; PubMed Central PMCID: PMC4452115. 23875796 PMC4452115

[11] Cole C, Petree LE, Phillips JP, Shoemaker JM, Holdsworth M, Helitzer DL. ’Ethical responsibility’ or ’a whole can of worms’: differences in opinion on incidental finding review and disclosure in neuroimaging research from focus group discussions with participants, parents, IRB members, investigators, physicians and community members. Journal of medical ethics. 2015;41(10):841–7. Epub 2015/06/13. doi: 10.1136/medethics-2014-102552 . 26063579

[12] Schmidt CO, Hegenscheid K, Erdmann P, Kohlmann T, Langanke M, Volzke H, et al. Psychosocial consequences and severity of disclosed incidental findings from whole-body MRI in a general population study. European radiology. 2013;23(5):1343–51. Epub 2012/12/15. doi: 10.1007/s00330-012-2723-8 . 23239059

[13] Rancher CE, Shoemaker JM, Petree LE, Holdsworth M, Phillips JP, Helitzer DL. Disclosing neuroimaging incidental findings: a qualitative thematic analysis of health literacy challenges. BMC medical ethics. 2016;17(1):58. Epub 2016/10/12. doi: 10.1186/s12910-016-0141-1 ; PubMed Central PMCID: PMC5057374. 27724936 PMC5057374

[14] Kaphingst KA, Ivanovich J, Elrick A, Dresser R, Matsen C, Goodman MS. How, who, and when: preferences for delivery of genome sequencing results among women diagnosed with breast cancer at a young age. Molecular genetics & genomic medicine. 2016;4(6):684–95. Epub 2016/11/30. doi: 10.1002/mgg3.254 ; PubMed Central PMCID: PMC5118211. 27896289 PMC5118211

[15] Kullo IJ, Haddad R, Prows CA, Holm I, Sanderson SC, Garrison NA, et al. Return of results in the genomic medicine projects of the eMERGE network. Frontiers in genetics. 2014;5:50. Epub 2014/04/12. doi: 10.3389/fgene.2014.00050 ; PubMed Central PMCID: PMC3972474. 24723935 PMC3972474

[16] Hartz SM, Olfson E, Culverhouse R, Cavazos-Rehg P, Chen L-S, DuBois J, et al. Return of individual genetic results in a high-risk sample: enthusiasm and positive behavioral change. Genetics in medicine: official journal of the American College of Medical Genetics. 2015;17(5):374–9. doi: 10.1038/gim.2014.110 PubMed PMID: PMC4344933. 25166427 PMC4344933

[17] Coors ME, Raymond KM, McWilliams SK, Hopfer CJ, Mikulich-Gilbertson SK. Adolescent perspectives on the return of individual results in genomic addiction research. Psychiatric genetics. 2015;25(3):127–30. Epub 2015/03/10. doi: 10.1097/YPG.0000000000000083 ; PubMed Central PMCID: PMC5094179. 25748091 PMC5094179

[18] De Clercq E, Kaye J, Wolf SM, Koenig BA, Elger BS. Returning Results in Biobank Research: Global Trends and Solutions. Genetic testing and molecular biomarkers. 2017;21(3):128–31. Epub 2017/02/02. doi: 10.1089/gtmb.2016.0394 ; PubMed Central PMCID: PMC5367909. 28146646 PMC5367909

[19] Mathers CD, Loncar D. Projections of global mortality and burden of disease from 2002 to 2030. PLoS medicine. 2006;3(11):e442. Epub 2006/11/30. doi: 10.1371/journal.pmed.0030442 ; PubMed Central PMCID: PMC1664601. 17132052 PMC1664601

[20] Corbin JSA. Basics of qualitative research: techniques and procedures for developing grounded theory. 3rd ed. Thousand Oaks (CA): Sage Publications; 2008.

[21] O’Daniel J, Haga SB. Public perspectives on returning genetics and genomics research results. Public health genomics. 2011;14(6):346–55. Epub 2011/05/11. doi: 10.1159/000324933 ; PubMed Central PMCID: PMC3221258. 21555865 PMC3221258

[22] Bollinger JM, Scott J, Dvoskin R, Kaufman D. Public preferences regarding the return of individual genetic research results: findings from a qualitative focus group study. Genetics in medicine: official journal of the American College of Medical Genetics. 2012;14(4):451–7. Epub 2012/03/10. doi: 10.1038/gim.2011.66 ; PubMed Central PMCID: PMC3927946. 22402755 PMC3927946

[23] Sanderson SC, Linderman MD, Suckiel SA, Diaz GA, Zinberg RE, Ferryman K, et al. Motivations, concerns and preferences of personal genome sequencing research participants: Baseline findings from the HealthSeq project. European journal of human genetics: EJHG. 2016;24(1):14–20. Epub 2015/06/04. doi: 10.1038/ejhg.2015.118 ; PubMed Central PMCID: PMC4795230. 26036856 PMC4795230

[24] Wong CA, Hernandez AF, Califf RM. Return of Research Results to Study Participants: Uncharted and Untested. JAMA. 2018;320(5):435–6. Epub 2018/06/23. doi: 10.1001/jama.2018.7898 . 29931289

[25] In: Downey AS, Busta ER, Mancher M, Botkin JR, editors. Returning Individual Research Results to Participants: Guidance for a New Research Paradigm. Washington (DC)2018. 30001048

[26] Halbert CH, Armstrong K, Gandy OH Jr., Shaker L. Racial differences in trust in health care providers. Archives of internal medicine. 2006;166(8):896–901. Epub 2006/04/26. doi: 10.1001/archinte.166.8.896 . 16636216

[27] Armstrong K, Rose A, Peters N, Long JA, McMurphy S, Shea JA. Distrust of the health care system and self-reported health in the United States. Journal of general internal medicine. 2006;21(4):292–7. Epub 2006/05/12. doi: 10.1111/j.1525-1497.2006.00396.x ; PubMed Central PMCID: PMC1484714. 16686803 PMC1484714

[28] Strong KA, Zusevics KL, Bick D, Veith R. Views of primary care providers regarding the return of genome sequencing incidental findings. Clinical genetics. 2014;86(5):461–8. Epub 2014/03/29. doi: 10.1111/cge.12390 . 24673592

[29] Middleton A, Morley KI, Bragin E, Firth HV, Hurles ME, Wright CF, et al. Attitudes of nearly 7000 health professionals, genomic researchers and publics toward the return of incidental results from sequencing research. European journal of human genetics: EJHG. 2016;24(1):21–9. Epub 2015/04/30. doi: 10.1038/ejhg.2015.58 ; PubMed Central PMCID: PMC4795240. 25920556 PMC4795240

[30] Vassy JL, Lautenbach DM, McLaughlin HM, Kong SW, Christensen KD, Krier J, et al. The MedSeq Project: a randomized trial of integrating whole genome sequencing into clinical medicine. Trials. 2014;15:85. Epub 2014/03/22. doi: 10.1186/1745-6215-15-85 ; PubMed Central PMCID: PMC4113228. 24645908 PMC4113228

[31] Christensen KD, Vassy JL, Jamal L, Lehmann LS, Slashinski MJ, Perry DL, et al. Are physicians prepared for whole genome sequencing? a qualitative analysis. Clinical genetics. 2016;89(2):228–34. Epub 2015/06/18. doi: 10.1111/cge.12626 ; PubMed Central PMCID: PMC4683111. 26080898 PMC4683111

[32] Suckiel SA, Linderman MD, Sanderson SC, Diaz GA, Wasserstein M, Kasarskis A, et al. Impact of Genomic Counseling on Informed Decision-Making among ostensibly Healthy Individuals Seeking Personal Genome Sequencing: the HealthSeq Project. Journal of genetic counseling. 2016;25(5):1044–53. Epub 2016/02/24. doi: 10.1007/s10897-016-9935-z . 26898680

[33] Hitch K, Joseph G, Guiltinan J, Kianmahd J, Youngblom J, Blanco A. Lynch syndrome patients’ views of and preferences for return of results following whole exome sequencing. Journal of genetic counseling. 2014;23(4):539–51. Epub 2014/01/23. doi: 10.1007/s10897-014-9687-6 ; PubMed Central PMCID: PMC4451087. 24449059 PMC4451087

[34] Wilkins CH, Mapes BM, Jerome RN, Villalta-Gil V, Pulley JM, Harris PA. Understanding What Information Is Valued By Research Participants, And Why. Health affairs (Project Hope). 2019;38(3):399–407. Epub 2019/03/05. doi: 10.1377/hlthaff.2018.05046 ; PubMed Central PMCID: PMC6706772. 30830824 PMC6706772

[35] Holm IA, Iles BR, Ziniel SI, Bacon PL, Savage SK, Christensen KD, et al. Participant Satisfaction With a Preference-Setting Tool for the Return of Individual Research Results in Pediatric Genomic Research. Journal of empirical research on human research ethics: JERHRE. 2015;10(4):414–26. Epub 2015/09/18. doi: 10.1177/1556264615599620 . 26376753 PMC6686662

[36] Kaphingst KA, Ivanovich J, Biesecker BB, Dresser R, Seo J, Dressler LG, et al. Preferences for return of incidental findings from genome sequencing among women diagnosed with breast cancer at a young age. Clinical genetics. 2016;89(3):378–84. Epub 2015/04/15. doi: 10.1111/cge.12597 ; PubMed Central PMCID: PMC4856148. 25871653 PMC4856148

[37] Jamal L, Robinson JO, Christensen KD, Blumenthal-Barby J, Slashinski MJ, Perry DL, et al. When bins blur: Patient perspectives on categories of results from clinical whole genome sequencing. AJOB empirical bioethics. 2017;8(2):82–8. Epub 2017/09/28. doi: 10.1080/23294515.2017.1287786 . 28949844 PMC6647021

[38] Biesecker BB, Lewis KL, Umstead KL, Johnston JJ, Turbitt E, Fishler KP, et al. Web Platform vs In-Person Genetic Counselor for Return of Carrier Results From Exome Sequencing: A Randomized Clinical Trial. JAMA Intern Med. 2018;178(3):338–46. Epub 2018/01/23. doi: 10.1001/jamainternmed.2017.8049 ; PubMed Central PMCID: PMC5885925. 29356820 PMC5885925

[39] Wolf SM, Branum R, Koenig BA, Petersen GM, Berry SA, Beskow LM, et al. Returning a Research Participant’s Genomic Results to Relatives: Analysis and Recommendations. J Law Med Ethics. 2015;43(3):440–63. Epub 2015/10/21. doi: 10.1111/jlme.12288 ; PubMed Central PMCID: PMC4617203. 26479555 PMC4617203

[40] Fiscella K, Sanders MR. Racial and Ethnic Disparities in the Quality of Health Care. Annu Rev Public Health. 2016;37:375–94. Epub 2016/01/21. doi: 10.1146/annurev-publhealth-032315-021439 . 26789384

[41] In: Olsen LA, Aisner D, McGinnis JM, editors. The Learning Healthcare System: Workshop Summary. The National Academies Collection: Reports funded by National Institutes of Health. Washington (DC) 2007. 21452449

[42] Tomlinson T, De Vries R, Ryan K, Kim HM, Lehpamer N, Kim SY. Moral concerns and the willingness to donate to a research biobank. Jama. 2015;313(4):417–9. Epub 2015/01/28. doi: 10.1001/jama.2014.16363 ; PubMed Central PMCID: PMC4443895. 25626040 PMC4443895

