# Supplementary material for: Return of individual research results: What do participants prefer and expect?
Source: PLoS One. 2021 Jul 29;16(7):e0254153. doi: 10.1371/journal.pone.0254153 (PMC8320928; doi:10.1371/journal.pone.0254153)
Supplement: S1 File — (DOCX) [file pone.0254153.s001.docx]

**Supplement**

**Return of Individual Research Results: What do participants prefer and expect?**

## **Survey questions**

1. Overall, how satisfied are you with your experience in the Project Baseline study?
   - Very satisfied
   - Somewhat satisfied
   - Neither satisfied nor dissatisfied
   - Somewhat dissatisfied
   - Very dissatisfied
2. What do you like most about the Project Baseline study?
   - Please do not include personal or medical information that may identify you.
   - [Open text]
3. What do you dislike most about the Project Baseline study?
   - Please do not include personal or medical information that may identify you.
   - [Open text]
4. Indicate any aspects of the Project Baseline study that have not met your expectations so far. Please select all that apply.
   - Sleep Sensor
   - Site visit(s)
   - Study Watch
   - Project Baseline mobile app
   - Speed of getting my results back
   - Stool sample process
   - Communication from the Project Baseline team
   - Support/help options
   - Other
   - Nothing - everything has met my expectations so far
5. [Only if user selects “Other” above] Please specify which aspect(s) of the Project Baseline study have not met your expectations so far.
   - Please do not include personal or medical information that may identify you.
   - [Open text]
6. Have you used the Project Baseline mobile app yet?
   - Yes
   - No
7. [Only if user selects “yes” above] How satisfied are you with the Project Baseline mobile app?
   - Very satisfied
   - Somewhat satisfied
   - Neither satisfied nor dissatisfied
   - Somewhat dissatisfied
   - Very dissatisfied
8. How confident are you that Project Baseline will keep your personal health information safe?
   - Extremely confident
   - Very confident
   - Moderately confident
   - Somewhat confident
   - Not at all confident
9. How confident are you that Project Baseline will provide adequate support if you have any problems or questions throughout the study?
   - Extremely confident
   - Very confident
   - Moderately confident
   - Somewhat confident
   - Not at all confident
10. Which results from your site visits would you be likely to share with a doctor or nurse if you got them back? Please select all that apply.
    - All of my results
    - Results I do not understand or have questions about
    - Abnormal results
    - Results my doctor or nurse is interested in having
    - Results that are relevant for an existing health condition(s)
    - Other
    - None of the above - I do not plan to share results with a doctor or nurse
    - N/A - I do not have access to a doctor or nurse
11. [Only if user selects “Other” above] Please specify which results from your site visits you would be likely to share with a doctor or nurse if you got them back.
    - Please do not include personal or medical information that may identify you.
    - [Open text]
12. Which of the following potential benefits is **most** appealing to you when receiving your results back, if any?
    - Have ownership of my data
    - Improve my health (either with help from a doctor or on my own)
    - Identify my risks for disease
    - Learn new or interesting things about my health
    - Other
    - None of the above - I do not want my results or do not care about these benefits
13. [Only if user selects “Other” above] Please specify what benefit is **most** appealing to you when receiving your results back.
    - Please do not include personal or medical information that may identify you.
    - [Open text]
14. When you think about getting your results from the site visits back, which statement best describes how you feel?
    - Excited to learn more about my health
    - Nervous about the possibility of receiving an abnormal result
    - Indifferent about getting my results back
    - Curious about the results
    - Other
    - None of the above
15. [Only if user selects “Other” above] Please specify how you feel when you think about receiving your results back.
    - Please do not include personal or medical information that may identify you.
    - [Open text]
16. You may have recently received your standard lab test results in the portal. Which of the following statements apply to you, if any? Please select all that apply.
    - I reviewed my results and do not have any questions
    - I did an online search or visited www.labtestsonline.org to better understand my results
    - I asked, or plan to ask, a doctor or nurse about my results
    - I contacted the Project Baseline team with questions about my results
    - Other
    - N/A - I have either not reviewed or not received my standard lab tests results yet
17. [Only if user selects “Other” above] Please specify.
    - Please do not include personal or medical information that may identify you.
    - [Open text]
18. Which of the following results would you be **most** interested in getting back next?
    - Genetics
    - Microbiome (e.g., stool sample)
    - Vital signs (e.g., blood pressure, body mass index)
    - Physical assessments (e.g., balance tests, strength tests)
    - Cognitive assessments (e.g., depression or anxiety screen)
    - Heart imaging (e.g., chest x-ray, echocardiogram)
    - Eye imaging
    - Sleep Sensor data (e.g., hours slept each night)
    - Study Watch activity data (e.g., step count)
    - Other
    - None of the above
19. [Only if user selects “Other” above] Please specify which results you would be **most** interested in getting back next.
    - Please do not include personal or medical information that may identify you.
    - [Open text]
20. In a typical week, on how many days do you wear your Study Watch?
    - 0
    - 1 to 2
    - 3 to 4
    - 5 to 6
    - 7
21. [Only if user selects any answer other than “7” above] What is the **main** reason you have not worn your Study Watch on certain days?
    - Forget to charge or sync the device
    - Forget to put the device on
    - Comfort issues
    - Appearance issues
    - Device data is not useful
    - I have another watch that I prefer
    - I have another wearable device (e.g., Apple Watch, Fitbit) that I prefer
    - Device was broken
    - Other
22. [Only if user selects “Other” above] Please specify the **main** reason you do not wear your Study Watch every day.
    - Please do not include personal or medical information that may identify you.
    - [Open text]
23. Overall, how satisfied are you with your Study Watch so far?
    - Very satisfied
    - Somewhat satisfied
    - Neither satisfied nor dissatisfied
    - Somewhat dissatisfied
    - Very dissatisfied

**Benchmark survey questions**

1. Which results from your site visits would you be likely to share with a doctor or nurse if you got them back?

Please select all that apply.

- - All of my results
- Results I do not understand or have questions about
- Abnormal results
- Results my doctor or nurse is interested in having
- Results that are relevant for an existing health condition(s)
- Other
- None of the above - I do not plan to share results with a doctor or nurse
- N/A - I do not have access to a doctor or nurse

1. [Only if user selects “Other” above] Please specify which results from your site visits you would be likely to share with a doctor or nurse if you got them back.
   - [hint text: Please do not include personal or medical information that may identify you.]
   - [opent text]

1. Which of the following potential benefits is most appealing to you when receiving your results back, if any?

- Have ownership of my data
- Improve my health (either with help from a doctor or on my own)
- Identify my risks for disease
- Learn new or interesting things about my health
- Other
- None of the above - I do not want my results or do not care about these benefits

1. [Only if user selects “Other” above] Please specify what benefit is most appealing to you when receiving your results back.
   - [hint text: Please do not include personal or medical information that may identify you.]
   - [Open text]

1. When you think about getting your results from the site visits back, which statement best describes how you feel?

- Excited to learn more about my health
- Nervous about the possibility of receiving an abnormal result
- Indifferent about getting my results back
- Curious about the results
- Other
- None of the above

1. [Only if user selects “Other” above] Please specify how you feel when you think about receiving your results back.
   - [hint text: Please do not include personal or medical information that may identify you.]
   - [Open text]

1. We recently returned your standard lab test results in the portal. Which of the following statements apply to you, if any?

Please select all that apply.

- I reviewed my results and do not have any questions
- I did an online search or visited www.labtestsonline.org to better understand my results
- I asked, or plan to ask, a doctor or nurse about my results
- I contacted the Project Baseline team with questions about my results
- Other
- N/A - I have either not reviewed or not received my standard lab tests results yet

1. [Only if user selects “Other” above] Please specify.
   - [hint text: Please do not include personal or medical information that may identify you.]
   - [Open text]

1. Which of the following results would you be most interested in getting back next?

- Genetics
- Microbiome (e.g., stool sample)
- Vital signs (e.g., blood pressure, body mass index)
- Physical assessments (e.g., balance tests, strength tests)
- Cognitive assessments (e.g., depression or anxiety screen)
- Heart imaging (e.g., chest x-ray, echocardiogram)
- Eye imaging
- Sleep Sensor data (e.g., hours slept each night)
- Study Watch activity data (e.g., step count)
- Other
- None of the above

1. [Only if user selects “Other” above] Please specify which results you would be most interested in getting back next.
   - [hint text: Please do not include personal or medical information that may identify you.]
   - [Open text]

**Data Dictionary**

| **VARIABLE** | **DESCRIPTION** | **DATA TYPE** | **CODES** |
| --- | --- | --- | --- |
| SEX | “What was your sex at birth?” | Character | Male Female |
| age_at_enrollment | Age at enrollment | Float |  |
| Site | Study site | Character | Durham Kannapolis Los Angeles Palo Alto |
| putative_cohort | Putative cohort | Character | PUTATIVE_COHORT_LOW_RISK PUTATIVE_COHORT_HIGH_RISK_CARDIO PUTATIVE_COHORT_HIGH_RISK_LUNG_CANCER PUTATIVE_COHORT_HIGH_RISK_OVARIAN_BREAST_CANCER |
| RACE | Which race do you identify with the most? | Character | American Indian or Alaska Native Asian Black or African American Native Hawaiian or Other Pacific Islander White Other |
| hispanic_ancestry | “Are you of Hispanic, Latino, or Spanish origin?” | Character | Yes No I don't know |
| circumstances_and_habits_income | Income | Character | Less than $10,000 $10,000- $24,999 $25,000- $34,999 $35,000- $49,999 $50,000- $74,999 $75,000-$99,999 $100,000- $149,999 $150,000- $199,999 $200,000 or more Prefer not to answer |
| circumstances_and_habits_highest_education | Highest Education | Character | Never attended school or only attended kindergarten Grades 1 through 4 (Primary) Grades 5 through 8 (Secondary) Grades 9 through 11 (Some high school) Grade 12 or GED (High school graduate) College 1 to 3 years (Some college or technical school) College 4 years or more (College graduate) Advanced degree (Master's, Doctorate, etc.) Prefer not to answer |
